# EVscope: A Comprehensive Bioinformatics Pipeline for Accurate and Robust Analysis of Total RNA Sequencing from Extracellular Vesicles

**DOI:** 10.1101/2025.06.24.660984

**Published:** 2025-06-27

**Authors:** Yiyong Zhao, Himanshu Chintalapudi, Ziqian Xu, Weiqiang Liu, Yuxuan Hu, Ewa Grassin, Minsun Song, SoonGweon Hong, Luke P. Lee, Xianjun Dong

**Affiliations:** 1 Stephen & Denise Adams Center for Parkinson’s Disease Research of Yale School of Medicine, New Haven, CT 06510, USA; 2 Department of Neurology, Yale School of Medicine, Yale University, New Haven, CT 06510, USA; 3 Aligning Science Across Parkinson’s (ASAP) Collaborative Research Network, Chevy Chase, MD 20815, USA; 4 Department of Medicine, Brigham and Women’s Hospital, Harvard Medical School, Harvard University, Boston, MA, USA; 5 Department of Bioengineering, Department of Electrical Engineering and Computer Science, University of California at Berkeley, Berkeley, CA, USA

## Abstract

**Motivation:**

Extracellular vesicle (EV) RNA sequencing has emerged as a powerful approach for studying RNA biomarkers and intercellular communication. Nevertheless, the extremely low abundance, fragmented nature and ubiquitous tissue origin of EV RNAs, alongside potential contamination from co-isolated materials, such as free DNA and bacterial RNA, pose substantial analytical challenges. These complexities highlight a pressing need for a standardized, computational workflow that ensures robust quality control and EV RNA characterization.

**Results:**

Here, we present EVscope, an open-source bioinformatics pipeline designed specifically for processing EV RNA-seq datasets. EVscope employs an optimized genome-wide expectation-maximization (EM) algorithm that significantly improves multi-mapping read assignment at single-base resolution by effectively leveraging alignment scores (AS) and local read coverage, specifically tailored for fragmented and low-abundance EV RNAs. Notably, EVscope uniquely generates EM-based BigWig files for downstream analysis, a capability currently unavailable in existing EM-based BigWig quantification tools. The pipeline systematically integrates 27 major steps, including quality control, analysis of library structure, contamination assessment, read alignment, read strandedness detection, UMI-based deduplication, RNA quantification, genomic DNA (gDNA) contamination correction, cellular and tissue source inference and visualization with a comprehensive HTML report. EVscope incorporates a comprehensive, updated annotation covering 19 distinct RNA biotypes, encompassing protein-coding genes, lncRNAs, miRNAs, piRNAs, retrotransposons (LINEs, SINEs, ERVs), and additional non-coding RNAs (tRNAs, rRNAs, snoRNAs). Furthermore, it leverages two highly balanced circRNA detection algorithms for robust circular RNA identification. Notably, a downstream module enables the inference of the tissue/cellular origins of EV RNAs using bulk and single-cell RNA-seq reference datasets. EVscope is implemented as a convenient, single-command Bash pipeline leveraging Conda-managed standard software packages and custom scripts, ensuring reproducibility and straightforward deployment.

**Availability and implementation:**

Code, documentation, and tutorials are available at GitHub (https://github.com/TheDongLab/EVscope) and archived on Zenodo (https://zenodo.org/records/15577789).

## 1. Introduction

Extracellular vesicles (EVs) mediate intercellular communication primarily through the transfer of functional RNA molecules. These nanosized, membrane-bound structures are secreted by nearly all cell types and regulate numerous physiological and pathological processes (Yáñez-Mó, et al., 2015). Consequently, RNAs derived from EVs have emerged as promising biomarkers for various diseases (Kumar, et al., 2024), including cancer and neurodegenerative disorders, highlighting the growing importance of EV-RNA sequencing (RNA-seq) (Cheng and Hill, 2022). Nevertheless, technical challenges remain due to the inherent characteristics of EV RNAs, such as their extremely low abundance, high heterogeneity (Willms, et al., 2018), fragmented RNA, and susceptibility to contamination by genomic DNA during cell lysis, bacterial RNA, and co-isolated proteins, complicating their effective isolation, sequencing, expression quantification and downstream analyses (Ramirez, et al., 2018).

Standard RNA-seq bioinformatics pipelines, predominantly designed for abundant and high-quality cellular RNAs, inadequately handle the extremely low abundance, fragmented nature, and contamination-prone characteristics of EV RNAs. For instance, conventional poly-A selection sequencing preferentially enriches protein-coding transcripts, neglecting crucial non-polyadenylated RNAs abundant in EVs, such as miRNAs and lncRNAs. Furthermore, traditional approaches typically fail to adequately manage contamination risks and multiple mapping reads, potentially resulting in false-positive findings, missed valuable RNA species and reduced reliability. Therefore, specialized bioinformatics pipelines tailored to EV total RNA-seq are urgently required for accurate annotation, quantification, and interpretation of complex EV RNA profiles.

We systematically reviewed methodologies for EV isolation, RNA extraction, and library preparation, evaluating their strengths and limitations. Traditional EV isolation methods, such as ultracentrifugation, generally exhibit lower efficiency and higher variability. In contrast, newer approaches such as the Exodos system offer improved purity, size uniformity (30–200 nm), efficiency and reproducibility. For RNA extraction (Chen, et al., 2021), the optimized TRIzol method is advantageous for EV samples characterized by low RNA yield and fragmented RNA, effectively preserving RNA integrity and minimizing DNA contamination (Mateescu, et al., 2017; Prendergast, et al., 2018). Regarding RNA-seq library construction, we recommend the SMARTer Pico v3 kit, given its superior sensitivity, robustness, and compatibility with ultra-low-input RNA samples at picogram levels typical of EV preparations. This kit effectively captures diverse RNA species, including short, fragmented, and non-polyadenylated RNAs by leveraging strand-specific RNA library construction and unique molecular identifier (UMI) technologies, enabling comprehensive profiling of heterogeneous EV RNA populations with minimal bias and contamination. Detailed methodological comparisons are provided in the supplementary materials.

To address these analytical challenges (Miceli, et al., 2024), we developed EVscope, an end-to-end computational pipeline designed to automate and standardize EV total RNA-seq analyses. EVscope incorporates a novel genome-wide EM-based algorithm for quantifying multi-mapping reads and integrates comprehensive quality control measures, RNA classification, and downstream analytical components, including the inference of the cellular and tissues origins of EV RNAs. This comprehensive workflow significantly enhances the reproducibility, robustness and scalability of EV RNA-seq analyses, providing an efficient tool for investigating EV-derived RNA signatures.

## 2. Materials and methods

EVscope is designed as a fully automated pipeline that processes raw RNA-seq data from EV samples and generates a comprehensive set of quality control reports, expression data, and visualization outputs. Detailed comparisons of EV isolation methods (EXODUS system), RNA extraction efficiency (TRIzol-based vs. column-based methods), RNA library preparation (SMARTer Pico v3 kit), and quality control assessments (adapter trimming and read length distributions) are provided in Supplementary Figures S1–S4. EVscope performance was rigorously validated using publicly available EV RNA-seq datasets (Table S1), highlighting the pipeline’s capability of accurately handling adapter trimming and multi-mapping read assignment. All analysis parameters and software versions are documented in the reproducible Conda environment provided with the pipeline. The pipeline is structured into the following major steps:

### Quality control (QC) and UMI motif visualization

Quality control of both raw sequencing reads and final cleaned reads after UMI-adapter trimming was performed using FastQC (https://www.bioinformatics.babraham.ac.uk/projects/fastqc) to ensure high sequencing data quality for downstream analyses. Unique Molecular Identifier (UMI) motif composition was visualized using custom Python scripts and inspected for correctness and structural integrity. For human EV RNA-seq libraries, we recommend using the SMARTer^®^ Stranded Total RNA-Seq Kit v3 – Pico Input Mammalian (Takara Bio), where the first 14 nucleotides of Read2 contain a structured UMI sequence consisting of a UMI-linker-UMI-adapter motif, which includes adapter and linker sequences. Specifically, the constant presence and integrity of the UMI linker sequences within the reads were confirmed visually as part of high-quality validation for RNA-seq library construction.

### Read Contamination Assessment

Bacterial contamination from human sources is detected and filtered using BBMap (v39.15) (https://sourceforge.net/projects/bbmap), which partitions reads against bacterial reference genomes, including *Escherichia coli* and Mycoplasma species (NCBI genome: https://www.ncbi.nlm.nih.gov/home/genomes). Kraken2 (Wood, et al., 2019) is used for taxonomic classification of reads, providing an overview of microbial and viral contaminants. The results are visualized using Krona (Ondov, et al., 2011) interactive plots. To address ribosomal RNA (rRNA), which can constitute a major fraction of total RNA libraries, EVscope uses RiboDetector (Deng, et al., 2022) on downsampled reads to accurately quantify rRNA contamination, enhancing assessment precision.

### UMI processing, adapter and UMI-read through trimming.

A multi-stage trimming process is implemented to prepare reads for alignment. First, Unique Molecular Identifiers (UMIs) are extracted from Read2 using UMI-tools (Smith, et al., 2017) and appended to the read headers for subsequent deduplication. Second, standard sequencing adapters are trimmed using cutadapt (Martin, 2011). Third, a novel module (UMIAdapterTrimR1.py) specifically addresses the potential for UMI “read-through” artifacts, which can occur in short-fragment libraries by trimming UMI sequences that have been erroneously incorporated into the 3’ end of Read1. Finally, reads undergo quality trimming with cutadapt to remove low-quality bases, ensuring only high-quality reads proceed to alignment.

### Read strandedness detection

The strandedness specificity of the RNA-seq library is determined using a customized RSeQC-based script (Wang, et al., 2012). Leveraging this strandedness information, EVscope optionally quantifies reads from both RNA-derived (correct strand) and potential genomic DNA-derived (opposite strand) sources separately using featureCounts (Liao, et al., 2013). Subsequently, the read counts from the incorrect strand are then subtracted from the correct strand’s counts to produce a gDNA-corrected expression profile, a critical step for accurately analyzing low-input total RNA samples. RNA integrity and other library metrics are collected using Picard tools (https://broadinstitute.github.io/picard).

### Alignment and various types of linear RNA identifications

Reads are aligned to the human reference genome (hg38) using STAR (Dobin, et al., 2012) in a two-pass mode with parameters (e.g., --outFilterMultimapNmax 100, --chimSegmentMin 10) to enhance mapping accuracy for EV-derived RNA fragments. RNA species are annotated using a comprehensive set of annotation references. The primary gene annotation is based on GENCODE comprehensive v45, which is supplemented with piRNA annotations from piRBase (Wang, et al., 2022) and retrotransposon repetitive element annotations from UCSC genome browser by RepeatMasker (Chen, 2004) and Dfam (Hubley, et al., 2016). This integrated annotation allows for the classification and quantification of a wide array of RNA biotypes, including protein-coding genes, circRNAs, piRNAs, LINEs, SINEs, ERVs, and other non-coding RNAs. Read counts are assigned to genomic features using either featureCounts (Liao, et al., 2013) for uniquely mapping reads or optionally RSEM (Li and Dewey, 2011) for multi-mapping read quantification. Expression matrices are generated in TPM and CPM formats.

### Circular RNA identification

Circular RNA identification was performed using two complementary computational methods, CIRI2 and CIRCexplorer2, to balance sensitivity and specificity while reducing false positives. CIRI2 (Gao, et al., 2017) was utilized first, as it excels in identifying novel circRNAs without reliance on prior annotations, offering a balanced performance between sensitivity and precision. Subsequently, circRNA candidates were further refined using CIRCexplorer2 (Zhang, et al., 2016), a method leveraging the comprehensive human GENCODE annotations v45 (Harrow, et al., 2012) (https://www.gencodegenes.org/human) to enhance the annotation accuracy, reduce false positives, and improve confidence in circRNA characterization.

### Single-base resolution read coverage generation using expectation-maximization (EM) algorithm for multi-mapping read assignment

We developed an expectation-maximization (EM) algorithm-based approach to accurately assign multi-mapped sequencing reads at single-base resolution, iteratively refining fractional alignments based on alignment scores (AS) and local read coverage derived from both uniquely and multi-mapped reads until convergence. We developed a novel tool, which we named EMapper, to implement this expectation-maximization (EM) algorithm. The EM algorithm (EMapper) initializes the fractional assignments uniformly across all potential alignments and iteratively refines them based on reads coverages and alignment scores (AS) until convergence, producing strand-specific, single-base resolution read BigWig coverage files. Expression quantification is performed by calculating the Mean Per-base CPM (MCPM), providing robust normalization suitable for cross-sample and gene-level comparisons and downstream analyses such as differential expression and functional association studies.

### Expression quantification and RNA type characterization

EVscope generates RNA distribution plots and expression matrices, providing an overview of RNA biotype composition. BigWig tracks are generated to visualize RNA coverage for both uniquely mapped reads and multi-mapped reads corrected by an Expectation-Maximization (EM) algorithm.

### Cellular origin inference

To determine the likely source of EV RNAs, EVscope integrates the ARIC deconvolution algorithm (Zhang, et al., 2022), which robustly estimates cell-type proportions from bulk RNA-seq data through systematic marker selection. Users can input custom bulk or single-cell reference datasets or leverage built-in references with GTEx v10 (Lonsdale, et al., 2013) and the Human Brain Cell Atlas v1.0 (Siletti, et al., 2023) to infer the cellular origins of EV-derived RNAs.

### HTML final report generation

The final HTML report for EV RNA-seq analysis using EVscope was generated using R Markdown (Xie, et al., 2018). Interactive data visualization and tabular summaries were prepared using the DT package (https://rstudio.github.io/DT/), and formatted tables were generated using the kableExtra package (Zhu, 2021). The overall document structure and navigation were organized with the bookdown package (Xie, 2016), providing clear sectioning and interactive table-of-contents navigation. Project paths and reproducibility were managed by the here package (Müller, 2020). The final report includes tabbed sections, interactive tables allowing direct export of data, and embedded external reports (e.g., QC reports from FastQC and cutadapt).

## 3. Conclusion

EVscope has been tested on multiple EV RNA-seq datasets, demonstrating effective contamination filtering, RNA classification, and cellular origin inference. EVscope systematically generates 27 structured output directories corresponding to each major pipeline step, providing detailed and clearly organized results, including QC reports, expression data, BigWig coverage files, and interactive HTML reports. EVscope is implemented as a Bash script, allowing easy execution and custom modifications. The full source code and user documentation are available at GitHub (https://github.com/TheDongLab/EVscope) and archived on Zenodo (https://zenodo.org/records/15577789) under an MIT open-source license. EVscope provides an automated, scalable solution for analyzing total RNA-seq data from extracellular vesicles. By incorporating comprehensive quality control, contamination detection, broad RNA biotype classification, and cellular origin inference, EVscope addresses key challenges in EV RNA-seq research. This tool standardizes data processing workflows, enhances reproducibility, and facilitates the discovery of EV RNA biomarkers.

## Supplementary Material

1

Supplementary data

Supplementary data are available at Bioinformatics online.

## Figures and Tables

**Figure 1:**
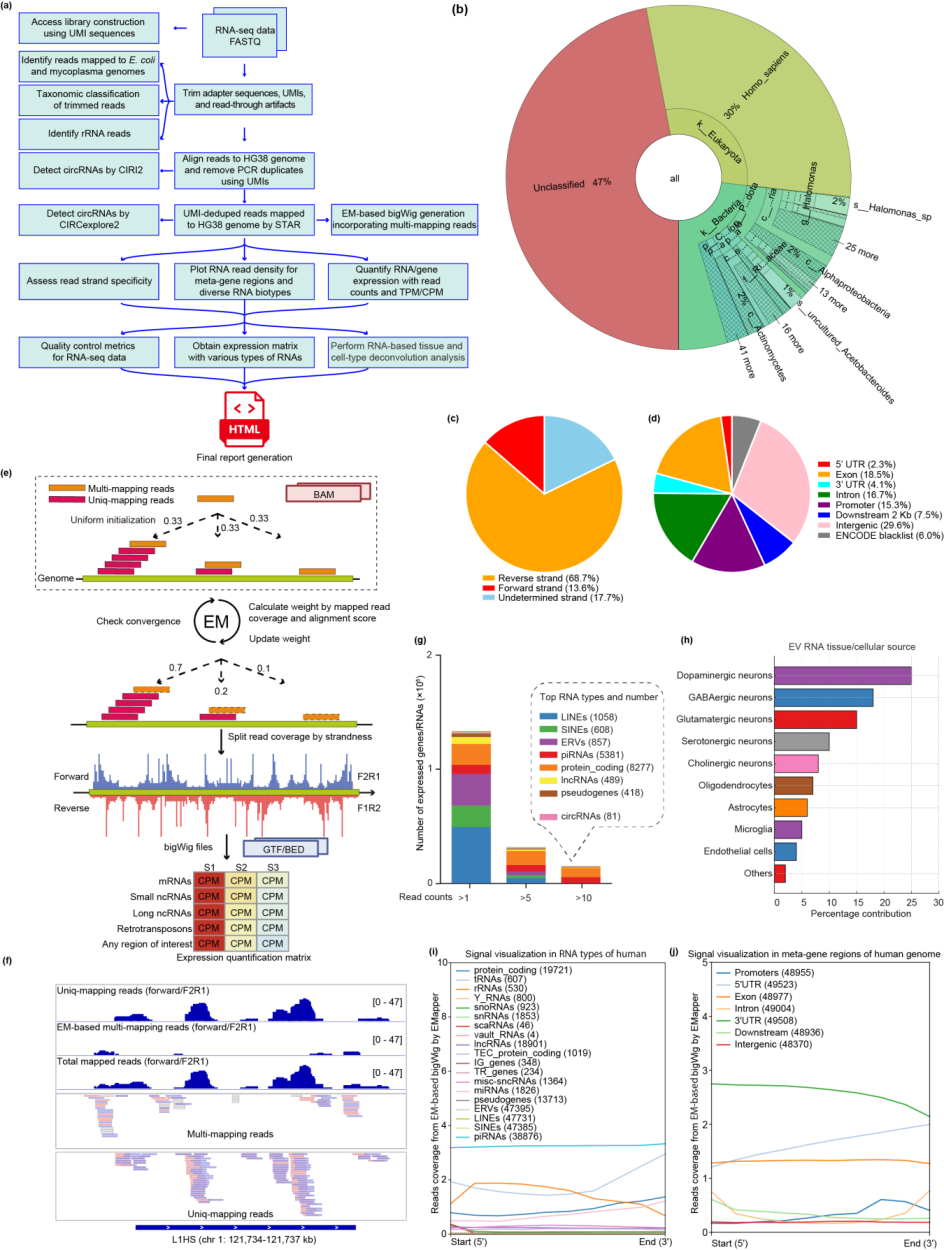
EVscope: A Comprehensive Bioinformatics Pipeline for Accurate and Robust Analysis of Total RNA Sequencing from Extracellular Vesicles. (a) Schematic overview of the EVscope bioinformatics workflow, highlighting key steps including quality control, contamination assessment, technical sequence trimming, read alignment, RNA quantification, EM-based BigWig generation and downstream analyses. (b) Taxonomic classification and distribution of EV RNA-seq reads using Kraken, visualized by a Krona plot highlighting predominant contaminants (e.g., bacteria) and unclassified reads. (c) Strand-specific analysis showing the distribution of read strand specificity. (d) Proportions of RNA-seq reads mapped to distinct genomic regions, represented by read count percentages. (e) Illustration of the Expectation-Maximization (EM) algorithm-based approach used by EVscope for accurate quantification and assignment of multi-mapped reads at single-base resolution, including a detailed example of BigWig tracks comparing uniquely mapped and EM-corrected multi-mapped reads. (f) Integrative Genomics Viewer (IGV) visualization example of EM algorithm-corrected BigWig tracks demonstrating enhanced resolution for multi-mapping read assignments. (g) Bar plot depicting the distribution and number of expressed RNAs across distinct biotypes within EV RNA-seq data. (h) Predicted cellular and tissue origins of EV-derived RNAs using single-cell RNA sequencing (scRNA-seq) deconvolution, displaying cell-type contributions ranked by abundance. (i) Visualization of EV RNA-seq signals across RNA biotypes in the human genome, highlighting biotype-specific EM-based read coverage patterns generated by EMapper. (j) Visualization of EV RNA-seq signals within meta-gene regions across the human genome, illustrating EM-based read coverage distribution across annotated genomic features by EMapper.

## Data Availability

Source code is available at GitHub (https://github.com/TheDongLab/EVscope) and archived on Zenodo (https://zenodo.org/records/15577789) under an MIT open-source license. Raw sequencing data used for validation are publicly available from NCBI Sequence Read Archive (SRA accession: SRR31350808–11). For any inquiries, please contact Dr. Xianjun Dong at xianjun.dong@yale.edu.
